# Glioblastoma post-operative imaging in neuro-oncology: current UK practice (GIN CUP study)

**DOI:** 10.1007/s00330-020-07387-3

**Published:** 2020-11-05

**Authors:** Thomas C. Booth, Aysha Luis, Lucy Brazil, Gerry Thompson, Rachel A. Daniel, Haris Shuaib, Keyoumars Ashkan, Anmol Pandey

**Affiliations:** 1 School of Biomedical Engineering & Imaging Sciences, King’s College London, London, SE1 7EH UK; 2 Department of Neuroradiology Ruskin Wing, King’s College Hospital NHS Foundation Trust, London, SE5 9RS UK; 3 Department of Neuroradiology, National Hospital For Neurology and Neurosrgery, London, WC1N 3BG UK; 4 Department of Oncology, Guy’s and St Thomas’ NHS Foundation Trust, London, SE1 7EH UK; 5 Centre for Clinical Brain Sciences, Edinburgh, EH16 4SB, UK; 6 Department of Medical Physics, Guy’s & St. Thomas’ NHS Foundation Trust, London, SE1 7EH UK; 7 Institute of Psychiatry, Psychology & Neuroscience, King’s College London, London, SE5 8AF UK; 8 Department of Neurosurgery, King’s College Hospital NHS Foundation Trust, London, SE5 9RS UK; 9 Faculty of Life Sciences and Medicine, King’s College London Strand, London, WC2R 2LS UK

**Keywords:** Glioblastoma, Neuroimaging, Guideline, Survey

## Abstract

**Objectives:**

MRI remains the preferred imaging investigation for glioblastoma. Appropriate and timely neuroimaging in the follow-up period is considered to be important in making management decisions. There is a paucity of evidence-based information in current UK, European and international guidelines regarding the optimal timing and type of neuroimaging following initial neurosurgical treatment. This study assessed the current imaging practices amongst UK neuro-oncology centres, thus providing baseline data and informing future practice.

**Methods:**

The lead neuro-oncologist, neuroradiologist and neurosurgeon from every UK neuro-oncology centre were invited to complete an online survey. Participants were asked about current and ideal imaging practices following initial treatment.

**Results:**

Ninety-two participants from all 31 neuro-oncology centres completed the survey (100% response rate). Most centres routinely performed an early post-operative MRI (87%, 27/31), whereas only a third performed a pre-radiotherapy MRI (32%, 10/31). The number and timing of scans routinely performed during adjuvant TMZ treatment varied widely between centres. At the end of the adjuvant period, most centres performed an MRI (71%, 22/31), followed by monitoring scans at 3 monthly intervals (81%, 25/31). Additional short-interval imaging was carried out in cases of possible pseudoprogression in most centres (71%, 22/31). Routine use of advanced imaging was infrequent; however, the addition of advanced sequences was the most popular suggestion for ideal imaging practice, followed by changes in the timing of EPMRI.

**Conclusion:**

Variations in neuroimaging practices exist after initial glioblastoma treatment within the UK. Multicentre, longitudinal, prospective trials are needed to define the optimal imaging schedule for assessment.

**Key Points:**

*• Variations in imaging practices exist in the frequency, timing and type of interval neuroimaging after initial treatment of glioblastoma within the UK.*

*• Large, multicentre, longitudinal, prospective trials are needed to define the optimal imaging schedule for assessment.*

**Supplementary Information:**

The online version of this article (10.1007/s00330-020-07387-3) contains supplementary material, which is available to authorized users.

## Introduction

Glioblastoma is the most common and aggressive primary malignant brain tumour in adults. It carries an annual incidence of 4.64 per 100,000 in England, with a peak between 65 and 75 years of age [1]. The current standard of care for newly diagnosed patients is maximal safe resection, followed by radiotherapy with concomitant and adjuvant temozolomide (TMZ) [2]. Despite this regimen, glioblastoma almost always recurs; the median overall survival is 14.6 months, whilst 5-year survival is below 10% [3].

Clinical review and serial neuroimaging remain the primary monitoring tools used to evaluate disease status and assess treatment response. However, on review of current UK, European and international guidelines, there is considerable variation and a paucity of evidence-based information regarding the optimal frequency, timing and type of neuroimaging following initial neurosurgical intervention [4–10]. Nevertheless, pragmatic neuroimaging time points are typically used in routine clinical practice which include an early post-operative MRI (EPMRI), a pre-radiotherapy MRI (PRMRI) and time points assessing the response of chemoradiotherapy both during and following completion of adjuvant TMZ [4–10].

EPMRI is frequently performed primarily to determine the extent of resection and to assess residual disease [11, 12]. There are recommendations that the EPMRI should be performed within 48–72 h due to the confounding effects of surgically induced contrast enhancement [11, 13]. The primary purpose for PRMRI is to delineate target volumes for radiotherapy planning, although this can also be achieved using fusion of the EPMRI with computed tomography (CT) in many cases [14].

Following completion of radiotherapy and concomitant TMZ, the first MRI examination is recommended to be performed 4–12 weeks subsequently [4, 7]. At this time point, approximately 20–30% of patients demonstrate a treatment-related effect, termed ‘pseudoprogression’ [15]. Pseudoprogression manifests as a transient increase in contrast enhancement and remains stable or eventually subsides without any change in treatment. Pseudoprogression appears to be the imaging manifestation of a subacute treatment-related tissue reaction which comprises inflammation, oedema, and increased permeability of the blood-brain barrier. The precise pathophysiological mechanism is still poorly understood, but histologic features typically associated with treatment effects such as bland necrosis with prominent vascular fibrinoid necrosis, reactive gliosis, oedema, demyelination and vascular hyalinisation are seen in those with pseudoprogression [16]. Pseudoprogression appears within 6 months of radiotherapy completion [17, 18], which is earlier than radiation necrosis [15, 19], another post-treatment-related effect (PTRE). Pseudoprogression appears to be more frequent in patients with a methylated MGMT gene promoter [15, 20].

Differentiating pseudoprogression from true progression has important implications in glioblastoma management but remains a major challenge as no standardised imaging biomarker has been definitively proven to be reliable [13]. In cases of suspected pseudoprogression, short-interval confirmatory MRI is recommended within 4–6 weeks whilst adjuvant TMZ treatment is continued [4, 9]. However, conventional structural imaging alone is often insufficient and unreliable. Advanced MR imaging techniques, such as perfusion imaging (dynamic susceptibility contrast-enhanced, DSC), permeability imaging (dynamic contrast-enhanced, DCE), ^1^H-magnetic resonance spectroscopy (MRS) and position emission tomography (PET) using radiolabelled amino acid tracers (l-[methyl-^11^C]methionine [MET], ^18^F-fluoroethyl-tyrosine [FET], ^18^F-fluoro-l-dihydroxy-phenylalanine [FDOPA] and ^11^C-alpha-methyl-l-tryptophan [AMT]), can provide additional physiological and metabolic information which may be helpful in distinguishing tumour progression from pseudoprogression [21–41].

In cases where tumour progression is confirmed with neuroimaging, management typically consists of second-line chemotherapy including the combination of procarbazine, lomustine and vincristine (PCV) [9, 42, 43], TMZ re-challenge [44, 45] or supportive care. There is less evidence to support re-irradiation and second surgery [9, 46, 47]. The specific strategy used depends on factors including performance status, risk of disability and prior treatment. In the absence of any tumour progression, a further MRI examination is generally recommended at the end of adjuvant TMZ [4], which serves as a new baseline for subsequent MRI examinations at 3–4 monthly intervals [9].

As the majority of the above recommendations were motivated by a need for reference standards in neuro-oncology clinical trials [48], we suspected that real-world neuro-oncology follow-up imaging practices vary considerably between UK centres. Given the lack of reliable and detailed data on current practice, we have surveyed all UK neuro-oncology centres to determine how neuroimaging is currently being used in the management of glioblastoma. Importantly, the survey will provide baseline data which is required to inform the design of studies aimed to optimise follow-up MRI imaging practice.

## Methods

### Participants

The UK Research Ethics Service (RES) provided written confirmation that ethical approval was not necessary for this study. Eligible participants were neuro-oncologists, neuroradiologists and neurosurgeons from all thirty-one neuro-oncology centres within the UK (public National Health Service academic centres). The participants were specialty leads or joint leads for their respective neuro-oncological service. As no national database of these neuro-oncology experts exists, we therefore identified potential participants’ contact details through institution websites and by liaison with relevant learned societies and known experts.

### Survey design

The survey featured forty-three questions, divided into single choice, multiple choice and free text questions (Appendix 1). Participants were asked specialty-specific and cross-specialty questions regarding the current imaging practices following initial treatment for glioblastoma at their institution, as well as their opinion on ideal practice, which was defined as practice without time or cost constraint.

We used tailored design and bimodal methodology [49] to increase response rates and obtain high-quality feedback. The questionnaire was designed with feedback from a neuro-oncology charity (a member of UK’s James Lind Alliance Priority Setting Partnership who wish to establish the value and benefit of neuro-oncological interval imaging) [50] and improved thereafter following pilot testing by neuro-oncologists, neuroradiologists and neurosurgeons from two centres who were specialty leads for their respective neuro-oncological service. There were no concerns regarding recall bias; nonetheless, two elements of the design reduced this risk. First, every individual neuro-oncologist, neuroradiologist and neurosurgeon who was the specialty lead at their respective neuro-oncological service was asked to answer the questionnaire to give a collective centre response for the core questions. Second, only the specialty leads were selected to complete the questionnaire because typically they are involved in designing imaging protocols and are aware of annual audit findings.

An online survey tool, Survey Monkey (www.surveymonkey.com), was used for data collection. Each participant was invited to complete the online survey via e-mail. Multiple individualised follow-up emails were sent to ensure completion of the survey. Where we received data from both joint leads, the questionnaire which contained more detailed responses was used for final analysis.

### Statistical analysis

We compiled data from the completed surveys in Microsoft Excel (Microsoft Corp). SPSS (IBM Corp) was used for descriptive statistical analysis and to make comparisons between groups using the chi-squared test (Yates corrected). For smaller sample sizes, Fisher exact test was used. Statistical significance was set at *p* < 0.05.

## Results

### Subject characteristics

We identified 124 eligible participants from 31 centres; of these, 109 completed the survey. One duplicate response was removed. In twelve centres where joint leads from the same specialty responded, the questionnaire which contained the more detailed response was used for final analysis. There were no discrepancies in any of the joint responses. The final analysis comprised 92 respondents across 31 centres, including one neuro-oncologist who covered two centres. Thus, 100% centre and specialty lead response rates were achieved. The characteristics of survey respondents are summarised in Table 1.

**Table 1:** Background characteristics of 92 respondents from 31 neuro-oncology centres included for final analysis. All respondents were leads or joint leads for their respective neuro-oncological service. All respondents were UK consultant grade (i.e. independent practitioner)

Characteristic	Value, *n* (%)
Speciality of respondents	
Neuroradiologist	31 (34%)
Neurosurgeon	31 (34%)
Neuro-oncologist	30* (33%)
Location of respondents	
East Midlands	3 (3%)
East of England	3 (3%)
South-East	9 (10%)
London	18 (20%)
North-East England	6 (7%)
North-West England	11 (12%)
Northern Ireland	3 (3%)
Scotland	12 (13%)
South-West	6 (7%)
Wales	3 (3%)
West Midlands	9 (10%)
Yorkshire	9 (10%)
Number of newly diagnosed glioblastoma cases per centre per year	
0 < 50	3 (10%)
50 < 100	7 (22%)
100 < 150	13 (42%)
150 < 200	8 (26%)
> 200	0 (0%)
Time as consultant	
Years (median; range)	11; 1–29

*A single neuro-oncologist covered two centres

### EPMRI

**Fig. 1:**
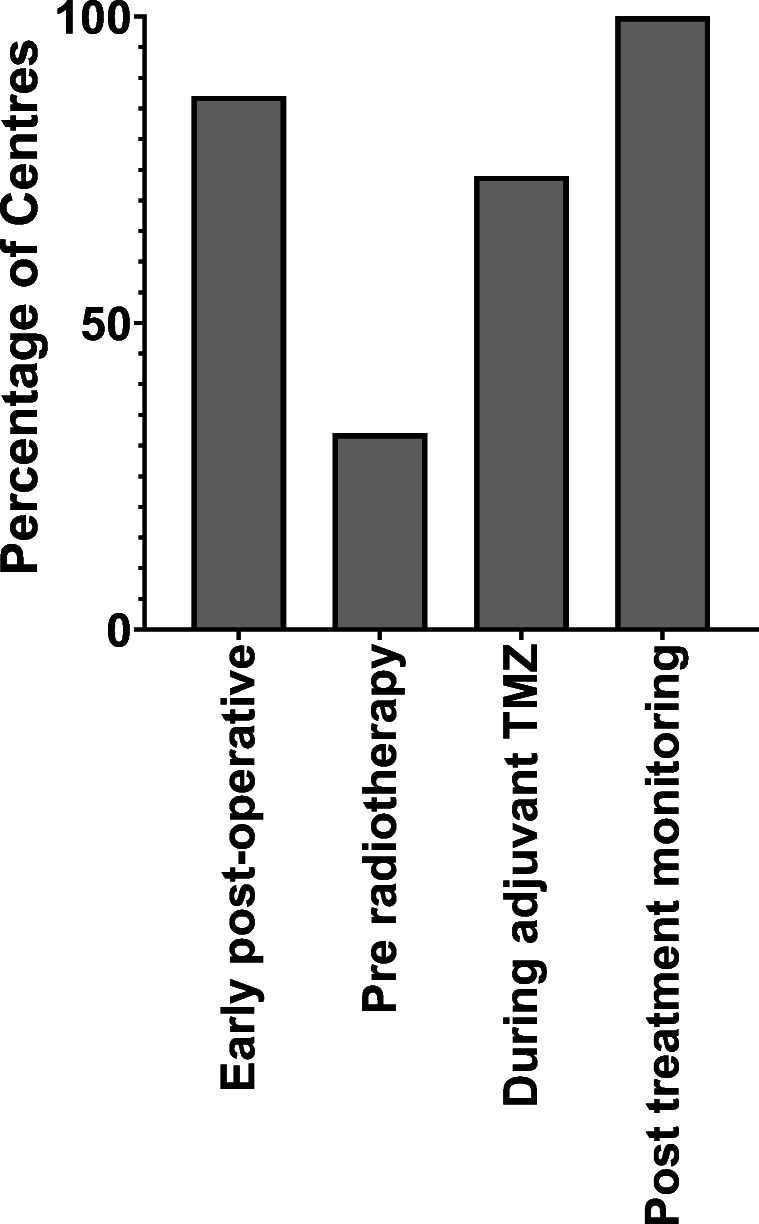
Percentage of centres that perform MRI at the different time points. All centres performed MRI after adjuvant TMZ, most centres performed MRI early post-operatively and during adjuvant TMZ treatment, whereas only 32% of centres routinely performed pre-radiotherapy MRI. TMZ = temozolomide

Most centres (87%, 27/31) reported that they routinely perform EPMRI (Fig. 1). The main reasons given for performing EPMRI were to quantify residual tumour volume and establish a baseline for subsequent examinations (Table 2). There were no significant differences in the rationale for EPMRI between specialties (*p* = 0.59).

**Table 2:** The rationale given for performing the early post-operative MRI (EPMRI) and pre-radiotherapy MRI (PRMRI)

Early post-operative MRI	Neuroradiologist	Neurosurgeon	Neuro-oncologist	Total
Purpose				
Establish amount of residual tumour	27	29	27	83 (90%)
Baseline to allow later assessment of treatment response	25	24	22	71 (77%)
Plan radiotherapy	12	14	21	47 (51%)
Differentiate between residual tumour and haemorrhage	16	13	14	43 (47%)
Differentiate between residual tumour and post-operative enhancement	15	11	14	40 (43%)
Differentiate between residual tumour and post-operative ischaemia	14	13	10	37 (40%)
Establish amount of residual tumour suitable for further resection	12	14	10	36 (39%) *p* = 0.59
Speciality specific				
EPMRI used to assess treatment response on later MRI	22 (71%)	–	–	
EPMRI used to decide on further debulking surgery	–	11 (35%)	–	
EPMRI used in the management of chemoradiotherapy	–	–	21 (70%)	
Pre-radiotherapy MRI				
Purpose				
Plan radiotherapy	9	6	10	25 (83%)
Baseline to allow later assessment of treatment response	7	2	3	12 (40%)
Establish amount of residual tumour	4	2	2	8 (27%)
Establish amount of residual tumour suitable for further resection	1	0	1	2 (7%) *p* = 0.74

In response to specialty-specific questions, EPMRI was reported to be used frequently by neuroradiologists and neuro-oncologists for purposes of treatment assessment and chemoradiotherapy planning respectively (71%, 22/31; 70%, 21/30). However, only a third of neurosurgeons (35%, 11/31) stated that the EPMRI formed an integral part of their decision-making process for further debulking surgery. It was reported that further debulking surgery was ultimately undertaken in only a small percentage (5–10%) of patients.

### PRMRI

Ten centres (32%, 10/31) reported that MRI was performed routinely prior to the commencement of radiotherapy (Fig. 1). A further eight centres (26%, 8/31) reported that co-registered CT was performed instead. In the remaining fourteen centres (45%, 14/31), it was unclear whether imaging was consistently performed at this time point. The most frequently reported reason for performing the PRMRI was radiotherapy planning (83%, 25/30). There were no significant differences in the rationale for PRMRI between specialties (Table 2; *p* = 0.74).

### MRI performed during adjuvant TMZ treatment

Twenty-three centres (74%, 23/31) reported that they had a standardised protocol for the adjuvant TMZ period (Fig. 1); three centres (10%, 3/31) avoided MRI during this period altogether; the remaining five centres did not provide a clear standardised protocol (16%, 5/31). The number and timing of scans routinely performed during adjuvant TMZ treatment varied widely (Table 3). Similarly, the standardised protocols implemented when there was suspected progression or new clinical symptoms also varied (Table 3). Most centres performed an MRI at the end of the adjuvant period (71%, 22/31).

**Table 3:** Distribution of the number and timing of scans routinely performed during adjuvant temozolomide (TMZ). Also shown are the standardised protocols implemented when there is suspected progression or new clinical symptoms

Number of scans during adjuvant period (not including post-cycle 6 TMZ)	Number of centres, *n* (%)
0	3 (10%)
1	7 (23%)
2	12 (39%)
3	4 (13%)
No standardised protocol	5 (16%)
Timing of first post-chemoradiotherapy scan	Number of centres, *n* (%)
4 weeks	12 (39%)
8 weeks	1 (3%)
12 weeks	10 (32%)
End of TMZ cycle 6 only	3 (10%)
No standardised protocol	5 (16%)
Protocol in suspected progression	Number of centres, *n* (%)
Additional imaging at 4–6 weeks	14 (45%)
Additional imaging over 4–12 weeks	8 (26%)
No standardised protocol	6 (19%)
No routine scans performed during adjuvant period	3 (10%)
Protocol if symptomatic during this period	Number of centres, *n* (%)
Initial CT, followed by MRI	11 (35%)
MRI	20 (65%)

### MRI performed after adjuvant TMZ treatment

All centres reported to have a standardised protocol for disease monitoring following the completion of adjuvant TMZ treatment (100%, 31/31; Fig. 1). Most centres performed MRI scans at 3 monthly intervals (81%, 25/31), although the length of follow-up was more variable (Table 4). In comparison to the adjuvant TMZ period, a significantly smaller proportion of centres reported having a standardised protocol to implement following suspected progression (11/31 vs 22/31, *p* = 0.019), opting instead for a more case-based approach (45%, 14/31).

**Table 4:** Distribution of the imaging intervals and duration of imaging after completion of adjuvant temozolomide (TMZ). Also shown is the standardised protocol implemented where there is suspected progression

Scan interval and duration	No. of centres, *n* (%)
3 monthly intervals for 6 months	3 (10%)
3 monthly intervals for 12 months	4 (13%)
3 monthly intervals for 18 months	1 (3%)
3 monthly intervals for 24 months	9 (29%)
3 monthly intervals for life	8 (26%)
4 monthly intervals for 18 months	2 (6%)
6 monthly intervals for life	4 (13%)
Protocol in suspected progression	No. of centres, *n* (%)
Additional imaging at 4–6 weeks	7 (23%)
Additional imaging at 6–12 weeks	4 (13%)
No standardised protocol	14 (45%)
No additional confirmatory scan	6 (19%)

### Assessing treatment response

Only 23% (7/31) of centres assessed treatment response based on the RANO criteria [13], whilst the MacDonald criteria [51] were not used at all (Table 5).

**Table 5:** Assessment of treatment response during and after adjuvant temozolomide (TMZ)

Assessment of treatment response	No. of centres, *n* (%)
MacDonald criteria based	0 (0%)
RANO criteria based	7 (23%)
Standardised protocol not based on either MacDonald or RANO criteria	12 (39%)
No standardised protocol	12 (39%)

### Imaging sequences and technique

**Fig. 2:**
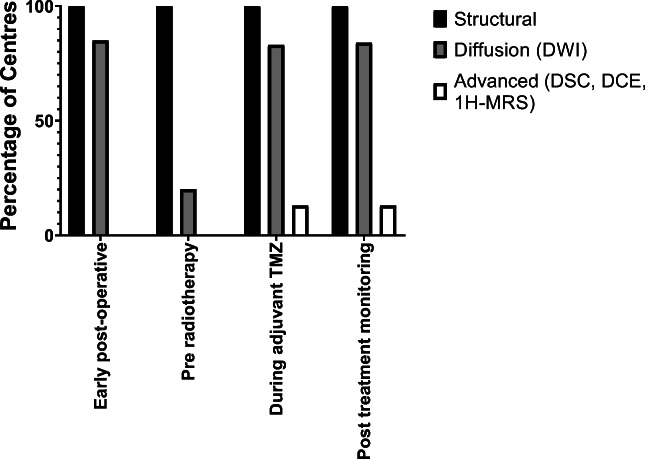
Imaging protocols at the different time points. Where the standardised protocol was described, most centres used diffusion weighted imaging (DWI), apart from at the pre-radiotherapy time point. Note that the figure shows the percentage of centres that routinely used advanced imaging techniques, as opposed to centres that only used advanced imaging techniques in selected patients, Structural = pre- and post-contrast T1-weighted, T2-weighted, FLAIR; DWI = diffusion weighted imaging; DSC = dynamic susceptibility contrast-enhanced MRI (perfusion); DCE = dynamic contrast enhanced MRI (permeability); ^1^H-MRS = ^1^H-MR spectroscopy

Structural sequences (pre- and post-contrast T1-weighted, T2-weighted, FLAIR) were used in all cases where the standardised protocol was described (Fig. 2). Diffusion weighted imaging (DWI) was used in the majority of EPMRI (85%, 23/27), during adjuvant TMZ (83%, 19/23) and after adjuvant TMZ (84%, 26/31; Fig. 2). Routine use of advanced imaging (DSC, DCE and/or MRS) on all patients was infrequent and limited to adjuvant TMZ and post-adjuvant TMZ periods (Fig. 2). Neuroradiologists from eleven centres (35%, 11/31), however, reported that advanced imaging was performed in selective cases to help differentiate between pseudoprogression and true progression during the adjuvant period ((*p* < 0.001). A further inconsistency between the specialties was seen during the EPMRI time point, whereby neuroradiologists reported more frequent use of volumetric imaging (*p* = 0.004).

### Ideal practice

In response to questions regarding ideal practice, thirty-seven respondents (40%, 37/92) reported that they would change their imaging protocol. Thirty-five respondents (38%, 35/92) did not suggest any changes, and the remaining twenty respondents (22%, 20/92) did not give a response.

Amongst those who suggested change in their current imaging protocol, approximately half (46%, 17/37) reported that adding advanced sequences such as DSC, DCE, MRS or PET would provide meaningful benefit, for subsequent comparison and to help differentiate between tumour progression and treatment-related changes. Although responses did not differ significantly between the three specialties questioned at EPMRI, PRMRI, and during and after adjuvant TMZ with regard to changing sequences (*p* = 0.1, *p* = 0.1, *p* = 0.6 and *p* = 0.8 respectively), we made three observations. Neuroradiologists were the main advocates for proposing advanced MRI during and following the adjuvant period (100%, 14/14). On the other hand, neuro-oncologists were more likely to suggest that further evidence is needed for analytical and clinical validation of these modalities in routine clinical practice (56%, 5/9). Conversely, neurosurgeons preferred to routinely add a volumetric acquisition to the EPMRI protocol to assess residual tumour volume (43%, 6/14; *p* = 0.04).

In terms of timing, it was noted that a small number of respondents (14%, 5/37), predominantly neurosurgeons, stated that they would want the EPMRI to be performed earlier than 72 h, whereas a minority of neuroradiologists stated that they would delay the timing to beyond 72 h to coincide with radiotherapy planning (8%, 3/37; *p* < 0.001). A further ideal practice suggestion was to ensure that EPMRI scans were available to all patients undergoing debulking surgery, including out of hours (27%, 10/37). Only a few respondents, predominantly neuro-oncologists, reported that they would add PRMRI (11%, 4/37; *p* = 0.7).

## Discussion

### Summary of findings

The GIN CUP study highlighted the variation in imaging practices in the treatment and follow-up periods of glioblastoma amongst the 31 neuro-oncological units in the UK. Over 80% of centres routinely performed EPMRI whereas only 32% performed PRMRI. During adjuvant TMZ treatment, there was considerable variation in the timing and frequency of imaging. There was more consistency at the end of the adjuvant period, at which point most centres (71%) performed MRI routinely, followed by monitoring scans at 3 monthly intervals (81%). The addition of advanced sequences was the most popular suggestion for ideal imaging practice, followed by changes in the timing of EPMRI.

### Comparison with other studies and study relevance

Over two decades ago, a prospective study showed that the volume of residual enhancing disease seen on EPMRI was an independent prognostic biomarker for both progression and overall survival [11]. This formed a rationale to perform EPMRI, which RANO subsequently advocated for use in trials in 2010 [13]. Compared to a 2010 survey of UK neuro-oncologists, our findings suggest an increase in the popularity of EPMRI in routine clinical practice (80% vs 50%) [4].

Our survey revealed that the most common reason for undertaking EPMRI was to determine the extent of residual tumour. However, recent studies have shown false-positive results where non-neoplastic enhancement appears within 72 h of surgery [52, 53]. Furthermore, 24% of cases appear to give false-negative results, as proven by histopathology or short-term follow-up [54]. In the recent era of TMZ chemotherapy and advanced neurosurgical techniques, a retrospective study demonstrated that EPMRI after tumour resection did not significantly affect overall survival [55]. In our study, the identification of residual disease infrequently altered subsequent management, as evidenced by neurosurgeons self-reporting that only 5–10% of patients undergo early repeat resection based on findings from EPMRI. This early repeat resection rate was 0% in a recent UK survey of 22 neurosurgical units, despite 16% (13/80) of cases being deemed operable [56]. It is possible that such evidence might explain why some centres choose not to perform EPMRI.

There is evidence that PRMRI could provide a more accurate baseline for subsequent imaging studies compared to EPMRI, due to the detection of any interval changes occurring between EPMRI and the initiation of chemoradiotherapy [57]. These changes include tumour growth [57–59] and new reactive non-neoplastic enhancement [11, 52, 53], both of which can confound treatment response assessment on subsequent imaging studies.

Pseudoprogression and radiation necrosis are two well-documented forms of PTRE. Pseudoprogression generally occurs within the six months following completion of chemoradiotherapy, and resolves or stabilises without additional treatment [15, 17–19, 60], whereas radiation necrosis generally occurs beyond 6 months, up to several years after radiotherapy, and is often more severe and progressive [15, 19]. Structural imaging alone cannot reliably discriminate between true progression and PTRE, due to the common features of contrast enhancement, perilesional oedema-like appearance and mass effect. Over the last two decades, there have been numerous promising studies to make this distinction, including the use of DSC, MRS and amino acid PET (Supplementary Table 1) [21–41, 61]. In our study, advanced techniques were used by only 10% of centres routinely and a third of centres in selected cases. This is in contrast to a survey of neuroradiologists across 220 European institutions in 2016, which reported routine use of advanced imaging for glioma follow-up (82% DSC, 80% MRS) [62]. However, the response rate for this European survey was 3%, and the results may not be representative of UK (or European) practice. The relatively low routine use of advanced imaging in our study might be related to the limited availability of expertise and software, as well as the increased operational costs, although many respondents suggested that advanced imaging techniques would improve their practice.

In our study, the largest variation in timing of MRI scans existed during the adjuvant period, which is comparable to a previous study [4]. In cases of suspected progression, only half of the centres emulated the 4–6 week “confirmatory” scan recommendation from RANO research guidelines [13]. One potential explanation for this is that two consecutive MRI within a short interval (4–6 weeks) may offer limited diagnostic value, particularly if they are performed during the enlargement phase of pseudoprogression [17]. Other limitations with imaging research guidelines have been described which might also have reduced their influence in a clinical setting [63].

Most centres used the same sequences at each of the different time points. The sequences included pre- and post-contrast T1-weighted, T2-weighted, FLAIR and DWI, apart from the PRMRI when DWI sequences were obtained infrequently. Consensus recommendations by a group of predominantly US experts for a standardised imaging protocol in trials [48] include pre- and post-contrast volumetric T1-weighted imaging, T2-weighted, FLAIR and DWI sequences. In our study, the use of volumetric imaging appeared to be underemployed; this was highlighted by neurosurgeons, who valued volumetric images as part of their ideal practice.

### Strengths and limitations

This was the first study to unequivocally capture UK neuro-oncology imaging practices by achieving a 100% response rate across three specialties from lead or joint lead consultants at all UK neuro-oncology centres. The 100% yield is important as it eliminated “nonresponse bias” due to an unrepresentative cohort response. The selection of these experts also eradicates “sampling bias” as these are the most expert clinicians for the subject matter. Our questionnaire was comprehensive and likely to capture the details of imaging practices following treatment for high-grade gliomas. There is benefit of including open-ended questions because they are not limited to a predetermined set of possible answer choices in order to extract more granular information. Indeed, after coding the granular information, a qualitative data analysis (QDA) provides useful information. However, although the questionnaire was designed with care and was improved after pilot testing, responses may reflect varying interpretation of the questions. It is also conceivable there may have been “recall bias”—after all, recall bias is almost impossible to entirely eradicate in surveys. However, the inclusion of experts who are typically involved in all aspects of neuro-oncology administration including attending weekly multi-disciplinary team meetings and planning departmental guidelines minimises recall bias. Additionally, questions regarding ideal practice only received responses from 50 to 60% of respondents distributed evenly across the three specialties. The reasons for poor response in this part of the survey are uncertain. Furthermore, although a health economic resource use analysis is beyond the remit of the study, it would have been interesting to explore resource use for each neuro-oncology centre, as it is possible this may have influenced imaging practices (Appendix 2). Our survey did not also address the emergence of novel therapies, such as immunotherapy and tumour-treating fields; however, these are not currently recommended as second-line treatment options in the UK outside of research trials.

### Unanswered questions and future directions

The variations in imaging practices elucidated by this study are most likely due to a lack of consensus and high-level evidence on the optimal schedule for imaging investigations during and after glioblastoma treatment. In particular, there has been no definitive study which addresses the question of how often MRI should be obtained in the post-treatment follow-up period. It is noteworthy that establishing the value and benefit of neuro-oncological interval imaging forms the second of ten priorities proposed by the UK’s James Lind Alliance Priority Setting Partnership, an organisation which aims to raise awareness for important research questions [50]. Our study records current clinical practice and highlights that there is variation between centres. We reiterate that the study does not aim to present optimal practice. To achieve optimal practice in this heterogeneous patient pathway where there are multiple co-variates, large, multicentre, longitudinal, prospective trials, possibly informed by data-driven machine learning algorithms [64], are now needed. Such studies could define the optimal time points for assessment and determine whether neuroimaging performed at each defined time point after initial glioblastoma treatment results in a real change in management and, more importantly, results in a change in patient outcomes such as morbidity and overall survival.

It is also noteworthy that if imaging could be performed without time or cost constraint, the expert community would add advanced sequences to current protocols. Mismatch between UK experts’ existing and perceived ideal MRI follow-up imaging regimens may reflect resource constraints and concerns over non-harmonised and non-validated advanced imaging protocols. This finding might motivate further development of cost-effective, harmonised and validated advanced sequences.

## Conclusion

The GIN CUP study assessed the current imaging practices amongst UK neuro-oncology centres and provided baseline data to inform future practice. We have shown definitively that variations in neuroimaging practices exist after initial glioblastoma treatment within the UK. Centre variation is unlikely to be in the best interests of all UK patients and is likely to reflect a lack of consensus and high-level evidence on the optimal schedule for imaging investigations during and after glioblastoma treatment. A validated post-operative imaging protocol with definitive evidence that outcomes are improved is now required. Multicentre, longitudinal, prospective trials interrogating protocols are recommended as is the development of efficient, harmonised and validated advanced sequences.

## Electronic supplementary material

ESM 1 (DOCX 3079 kb)

## Abbreviations

ADC: Apparent diffusion co-efficient
DCE: Dynamic contrast enhanced
DSC: Dynamic susceptibility contrast enhanced
DTI: Diffusion tensor imaging
DWI: Diffusion weighted imaging
EPMRI: Early post-operative MRI
FLAIR: Fluid-attenuated inversion recovery
MGMT: O^6^-Methylguanine-DNA methyltransferase
MRS: Magnetic resonance spectroscopy
PCV: Procarbazine, lomustine and vincristine
PET: Positron emission tomography
PRMRI: Pre-radiotherapy MRI
RANO: Response Assessment in Neuro-Oncology
SWI: Susceptibility weighted imaging
TMZ: Temozolomide
WHO: World Health Organization
