# Supplementary material for: Glioblastoma post-operative imaging in neuro-oncology: current UK practice (GIN CUP study)
Source: Eur Radiol. 2020 Nov 5;31(5):2933–43. doi: 10.1007/s00330-020-07387-3 (PMC8043861; doi:10.1007/s00330-020-07387-3)
Supplement: Supplementary file 1 — (DOCX 3079 kb) [file 330_2020_7387_MOESM1_ESM.docx]

Supplementary Table: a summary of the advanced imaging findings suggestive of true progression or pseudoprogression.

|  | **True Progression*** | **Pseudoprogression*** |
| --- | --- | --- |
| Dynamic Susceptibility Contrast (DSC) MR Perfusion [1-5] | Increased rCBV | Reduced or normal rCBV |
| Dynamic Contrast Enhanced (DCE) MR Perfusion [4-6] | Increased K^trans^ and Vp | Reduced or normal K^trans^ and Vp |
| 1H-MR Spectroscopy [5, 7-9] | Higher Choline/NAA ratio  Presence of lipid-lactate peak | Lower choline/NAA ratio |
| ^11^C MET PET [10]  O-(2-[^18^F]fluoroethyl)-L-tyrosine (^18^F–FET)[11, 12] | Higher SUV_max_/SUV_mean_ /TBR_max_/ TBR_mean_ | Reduced or normal SUV_max_/SUV_mean_ /TBR_max_/ TBR_mean_ |

rCBV = relative cerebral blood volume

K^trans^ = volume transfer constant

Vp = volumetric plasma volume

NAA = N-acetylaspartate

SUV = standardised uptake value

TBR = tumour-to-brain ratio

*The thresholds used will vary according to the imaging protocol and software used for analysis [1-12].

References

1. Sugahara, T., et al., *Posttherapeutic intraaxial brain tumor: the value of perfusion-sensitive contrast-enhanced MR imaging for differentiating tumor recurrence from nonneoplastic contrast-enhancing tissue.* AJNR Am J Neuroradiol, 2000. **21**(5): p. 901-9.

2. Hu, L.S., et al., *Relative cerebral blood volume values to differentiate high-grade glioma recurrence from posttreatment radiation effect: direct correlation between image-guided tissue histopathology and localized dynamic susceptibility-weighted contrast-enhanced perfusion MR imaging measurements.* AJNR Am J Neuroradiol, 2009. **30**(3): p. 552-8.

3. Patel, P., et al., *MR perfusion-weighted imaging in the evaluation of high-grade gliomas after treatment: a systematic review and meta-analysis.* Neuro Oncol, 2017. **19**(1): p. 118-127.

4. Seeger, A., et al., *Comparison of three different MR perfusion techniques and MR spectroscopy for multiparametric assessment in distinguishing recurrent high-grade gliomas from stable disease.* Acad Radiol, 2013. **20**(12): p. 1557-65.

5. van Dijken, B.R.J., et al., *Diagnostic accuracy of magnetic resonance imaging techniques for treatment response evaluation in patients with high-grade glioma, a systematic review and meta-analysis.* Eur Radiol, 2017. **27**(10): p. 4129-4144.

6. Thomas, A.A., et al., *Dynamic contrast enhanced T1 MRI perfusion differentiates pseudoprogression from recurrent glioblastoma.* J Neurooncol, 2015. **125**(1): p. 183-90.

7. Zhang, H., et al., *Role of magnetic resonance spectroscopy for the differentiation of recurrent glioma from radiation necrosis: a systematic review and meta-analysis.* Eur J Radiol, 2014. **83**(12): p. 2181-2189.

8. Wang, Q., et al., *The diagnostic performance of magnetic resonance spectroscopy in differentiating high-from low-grade gliomas: A systematic review and meta-analysis.* Eur Radiol, 2016. **26**(8): p. 2670-84.

9. Kazda, T., et al., *Advanced MRI increases the diagnostic accuracy of recurrent glioblastoma: Single institution thresholds and validation of MR spectroscopy and diffusion weighted MR imaging.* Neuroimage Clin, 2016. **11**: p. 316-321.

10. Deuschl, C., et al., *(11)C-MET PET/MRI for detection of recurrent glioma.* Eur J Nucl Med Mol Imaging, 2018. **45**(4): p. 593-601.

11. Galldiks, N., et al., *Diagnosis of pseudoprogression in patients with glioblastoma using O-(2-[18F]fluoroethyl)-L-tyrosine PET.* Eur J Nucl Med Mol Imaging, 2015. **42**(5): p. 685-95.

12. Kebir, S., et al., *Late Pseudoprogression in Glioblastoma: Diagnostic Value of Dynamic O-(2-[18F]fluoroethyl)-L-Tyrosine PET.* Clin Cancer Res, 2016. **22**(9): p. 2190-6.

**Appendix 1**

**Questionnaire: Imaging adult patients during and following treatment for cerebral glioblastoma**

This survey is intended to capture the routine imaging performed for adult patients in your institution with newly diagnosed glioblastoma who have not been entered into clinical trials.

It should take approximately 15 minutes to complete. You can complete a portion of the survey and return later to finish it. Please try to answer all the questions.

A “glioblastoma” is defined here as a WHO grade IV cerebral astrocytoma.

An “EPMRI” is defined as the early post-operative MRI scan which is conducted between 24 hours to 72 hours post-operatively in patients undergoing glioblastoma debulking.

***DETAILS OF THE RESPONDER***

1. In which specialty are you a consultant?

Neuroradiology

Neurosurgery

Oncology

Other (please specify)

1. How many years have you been practising as a consultant?
2. Which region in the UK are you working in?
3. How many new patients with glioblastoma are managed in your centre every year?

0-50

50-100

100-150

150-200

200-250

>250

***SECTION A: EARLY POST-OPERATIVE MRI***

1. Does your institution perform an early post-operative MRI (EPMRI) on patients after debulking surgery for glioblastoma?

Yes

No

Not known

1. If known, approximately what percentage of glioblastoma patients have this scan?
2. If known, which MR technique and sequences are used? (please select as many as appropriate)

MR structural sequences (e.g. T1, T2 etc)

MR spectroscopy (single)

MR spectroscopy (multivoxel)

MR diffusion imaging (DWI/ADC)

MR diffusion imaging (DTI)

MR perfusion imaging (DSC)

MR permeability imaging (DCE)

1. If known, for structural sequences, please also state plane(s). Please select all that apply.

|  | Axial | Sagittal | Coronal | Volumetric |
| --- | --- | --- | --- | --- |
| T1 |  |  |  |  |
| T2 |  |  |  |  |
| FLAIR |  |  |  |  |
| T1 post contrast |  |  |  |  |

1. If known, why do you perform the EPMRI scan? (please select as many as appropriate)

To establish the amount of residual tumour

To differentiate between residual tumour and post-operative ischaemic lesions

To differentiate between residual tumour and post-operative changes such as post-operative enhancement

To differentiate between residual tumour and post-operative changes such as blood degradation products

To establish the amount of residual tumour suitable for further resection

To use as a baseline post-surgery, pre-radiotherapy/temozolomide scan to allow later assessment of treatment response

To plan radiotherapy

Other (please specify)

1. Neurosurgeons to answer only: Do findings from an EPMRI scan sometimes lead to further debulking surgery prior to adjuvant treatment?

Yes

No

Not known

1. If yes, what percentage approximately?
2. Neuro-oncologists to answer only: Do findings from the EPMRI have any bearing on chemoradiotherapy?

Yes

No

Not known

1. If Yes, please specify
2. Neuroradiologists to answer only: Do findings from the EPMRI help you to assess treatment response on later MRI?

Yes

No

Not known

Other (please specify)

1. Please mention any changes in the subsequent management/treatment which are not mentioned above
2. In an ideal world (i.e. without time or cost constraint) would you change any of your EPMRI imaging protocol (including MRI sequences or timing of scan) and if so, why is this?

***SECTION A – MRI PERFORMED AFTER 72 HOURS AND PRE-RADIOTHERAPY/TMZ***

1. Do you perform an MRI which is post surgery (after 72 hours) and pre radiotherapy/concomitant temozolomide (TMZ)?

Yes

No

Not known

1. If yes, what percentage (approximately) of glioblastoma patients have this MRI that is post surgery after 72 hours) and pre radiotherapy/TMZ?
2. If known, which MR technique and sequences are used for this post-surgical (after 72 hours) and pre-radiotherapy/TMZ MRI? (please select as many as appropriate).

MR structural sequences (e.g. T1, T2 etc)

MR spectroscopy (single)

MR spectroscopy (multivoxel)

MR diffusion imaging (DWI/ADC)

MR diffusion imaging (DTI)

MR perfusion imaging (DSC)

MR permeability imaging (DCE)

1. If known, for structural sequences, please also state plane(s). Please select all that apply.

|  | Axial | Sagittal | Coronal | Volumetric |
| --- | --- | --- | --- | --- |
| T1 |  |  |  |  |
| T2 |  |  |  |  |
| FLAIR |  |  |  |  |
| T1 post contrast |  |  |  |  |

1. Why do you perform this post-surgical (after 72 hours) and pre radiotherapy/TMZ MRI? (please select as many as appropriate)

To establish the amount of residual tumour

To establish the amount of residual tumour suitable for further resection

To use as a baseline post-surgery, pre-radiotherapy/temozolomide scan to allow later assessment of treatment response

To plan radiotherapy

Other (please specify)

1. In an ideal world (i.e. without time or cost constraint) would you change any of the imaging protocol post 72 h and pre radiotherapy/TMZ (including MRI sequences or timing of scan) and if so, why is this?
2. In the space below, please provide any further information which you feel may be relevant to the questions above

*(e.g. instead of an MRI we perform a CT post 72 hour and pre radiotherapy/TMZ for treatment planning. We fuse the EPMRI with this and use these as our final treatment planning images).*

***SECTION B – MRI PERFORMED POST RADIOTHERAPY/CONCOMITTANT TMZ DURING ADJUVANT TREATMENT***

1. Do you have a standard MRI protocol for routine post-radiotherapy/concomitant TMZ scanning (until the end of the adjuvant temozolomide period)?

Yes

No

1. If known, which MR technique and sequences are used for routine post-radiotherapy/concomitant TMZ scanning (until the end of the adjuvant temozolomide period)? (please select as many as appropriate)

MR structural sequences

MR spectroscopy (single)

MR spectroscopy (multivoxel)

MR diffusion imaging (DWI/ADC)

MR diffusion imaging (DTI)

MR perfusion imaging (DSC)

MR permeability imaging (DCE)

1. If known, for structural sequences, please also state plane(s). Please select all that apply.

|  | Axial | Sagittal | Coronal | Volumetric |
| --- | --- | --- | --- | --- |
| T1 |  |  |  |  |
| T2 |  |  |  |  |
| FLAIR |  |  |  |  |
| T1 post contrast |  |  |  |  |

1. If known, what is the standard follow-up imaging protocol at your institution from the end of radiotherapy/concomitant temozolomide treatment completion until the end of the adjuvant temozolomide period?

*(If tolerated, the typical adjuvant TMZ management is approximately 4 weeks rest after the end of radiotherapy/concomitant TMZ followed by 6 cycles of adjuvant TMZ. Each adjuvant TMZ cycle typically consists of 5 days treatment followed by 23 days of rest)*

Approximately 1 month post radiotherapy/concomitant TMZ treatment completion

Approximately 2 months post radiotherapy/concomitant TMZ treatment completion

Approximately 3 months post radiotherapy/concomitant TMZ treatment completion

Approximately 4 months post radiotherapy/concomitant TMZ treatment completion

Approximately 5 months post radiotherapy/concomitant TMZ treatment completion

Approximately 6 months post radiotherapy/concomitant TMZ treatment completion

1. Do you perform a confirmatory MRI scan (post radiotherapy/concomitant TMZ treatment completion during the adjuvant TMZ period) after a standard protocol time point to confirm/refute: (please select all answers that apply to your current routine practice)

Please note that the question below is linked to this question and reading the example may help you respond.

Complete response

Partial response

Stable disease

Progression

1. Of those selected above, how many weeks after a standard protocol time point do you perform a confirmatory MRI scan? Please explain each selection.

*(e.g. After seeing a disease progression we scan 4 weeks later to confirm disease progression).*

1. Do you perform an MRI scan if the patient becomes symptomatic (post radiotherapy/concomitant TMZ treatment completion during the adjuvant TMZ period)?

Yes

No

Other (please specify)

(e.g. *we always perform an immediate CT if the patient becomes symptomatic to look for neurosurgical emergencies such as haemorrhage. We typically proceed to MRI afterwards unless this is futile due to poor performance status).*

1. Please feel free to inform us of any further comments regarding use of additional MRI scans conducted during the adjuvant TMZ period

*(e.g. After seeing disease progression, we scan 4 weeks later and we add a perfusion scan (DSC) to our standard sequences. This helps us to either refute progression in the case of pseudoprogression or confirm progression).*

1. In an ideal world (i.e. without time or cost constraint) would you change any of the imaging protocol in the adjuvant TMZ period (including MRI sequences and timing of scans) and if so why is this?

*(e.g. On all our routine scans in the adjuvant period we would like to perform a perfusion scan (DSC) in addition to our standard sequences and also perform a methionine PET scan. This would help us to either refute progression, in the case of pseudoprogression, or confirm progression).*

***SECTION B – MRI AFTER THE ADJUVANT TMZ PERIOD***

1. If known, do you have a standard protocol for performing MRI following adjuvant TMZ completion (i.e. typically after 6 cycles of TMZ)?

Yes

No

1. If known, which MR technique and sequences are used? (please select as many as appropriate)

MR structural sequences

MR spectroscopy (single)

MR spectroscopy (multivoxel)

MR diffusion imaging (DWI/ADC)

MR diffusion imaging (DTI)

MR perfusion imaging (DSC)

MR permeability imaging (DCE)

1. If known, for structural sequences, please also state plane(s). Please select all that apply.

|  | Axial | Sagittal | Coronal | Volumetric |
| --- | --- | --- | --- | --- |
| T1 |  |  |  |  |
| T2 |  |  |  |  |
| FLAIR |  |  |  |  |
| T1 post contrast |  |  |  |  |

1. If known, what is your standard protocol for performing MRI scans following adjuvant TMZ completion (i.e. typically after 6 cycles of adjuvant TMZ)?

Please indicate the months at which the MRI scan is conducted and select ALL answers that apply to your current routine practice. Please select as many as appropriate.

1

2

3

4

5

6

7

8

9

10

11

12

13

14

15

16

17

18

19

20

21

22

23

24

>24

1. Please add comments below to further illustrate this protocol

*(e.g. following adjuvant TMZ completion our protocol is to conduct scans at six month intervals for the rest of the patient's life).*

1. Do you perform a confirmatory MRI scan (post adjuvant TMZ treatment completion) after a standard protocol time point to confirm/refute (please select all answers that apply to your current routine practice)

Please note that the question below is linked to this question and reading the example may help you respond.

Complete response

Partial response

Stable disease

Progression

1. Of those selected above, how many weeks after a standard protocol time point do you perform a confirmatory MRI scan? Please explain each selection

*(e.g. After seeing a disease progression we scan 4 weeks later to confirm disease progression)*

1. Do you perform an MRI scan if the patient becomes symptomatic (post adjuvant TMZ treatment completion)?

Yes

No

Other (please specify)

*(e.g. we always perform an immediate CT if the patient becomes symptomatic to look for neurosurgical emergencies such as haemorrhage. We typically proceed to MRI afterwards unless this is futile due to poor performance status).*

1. In an ideal world (i.e. without time or cost constraint) would you change any of the imaging protocol after the adjuvant TMZ period (including MRI sequences and timing of scan) and if so why is this?

*(e.g. On all our 6 monthly routine scans following the adjuvant period we would like to perform a perfusion scan (DSC) in addition to our standard sequences and also perform a methionine PET scan. This would help us to either refute progression, in the case of radiation necrosis, or confirm progression).*

***SECTION C – ASSESSING TREATMENT RESPONSE***

1. Do you follow the MacDonald or RANO glioblastoma treatment outcome criteria?

(Please select all answers that represent your current routine practice)

Yes. We follow the MacDonald criteria and it is the strict basis of our treatment response

Yes. We follow the MacDonald criteria and it is the approximate basis of our treatment response

Yes. We follow the RANO criteria and it is the strict basis of our treatment response

Yes. We follow the RANO criteria and it is the approximate basis of our treatment response

No. We follow neither

Unsure

Other (please elaborate and be specific as many centres use a mixture of influences and guidelines)

*(e.g. RANO outcome criteria are partially used for treatment response because we use RANO criteria in terms of querying progression when there is a > 25% increase in contrast enhancement or an increase in T2 hyperintensity but we do not use the recommendations about performing a confirmatory 4 week scan to confirm a partial or complete response. We also perform an immediate confirmatory perfusion scan (DSC) to confirm/refute progression rather than wait for confirmatory structural imaging 4 weeks later).*

1. For other high grade gliomas (such as anaplastic astrocytomas), would your answers to the above differ if the tumour was not a glioblastoma?

If so please provide free text on how it differs (please elaborate and be as specific as possible so that your scanning regimen can be fully understood).

*(e.g. we follow exactly the same imaging regimen as answered above for glioblastoma in terms of (i) EPMRI (ii) post 72 h surgery/pre radiotherapy and (iii) adjuvant chemotherapy period. However, the difference is that following the adjuvant chemotherapy period, routine MRI will be tailored to the prognostic profile of the tumour e.g. if this was an anaplastic oligodendroglioma with a good prognostic profile we would perform interval imaging every 9 months).*

(Please see Appendix 3 for the response to question 43)

*
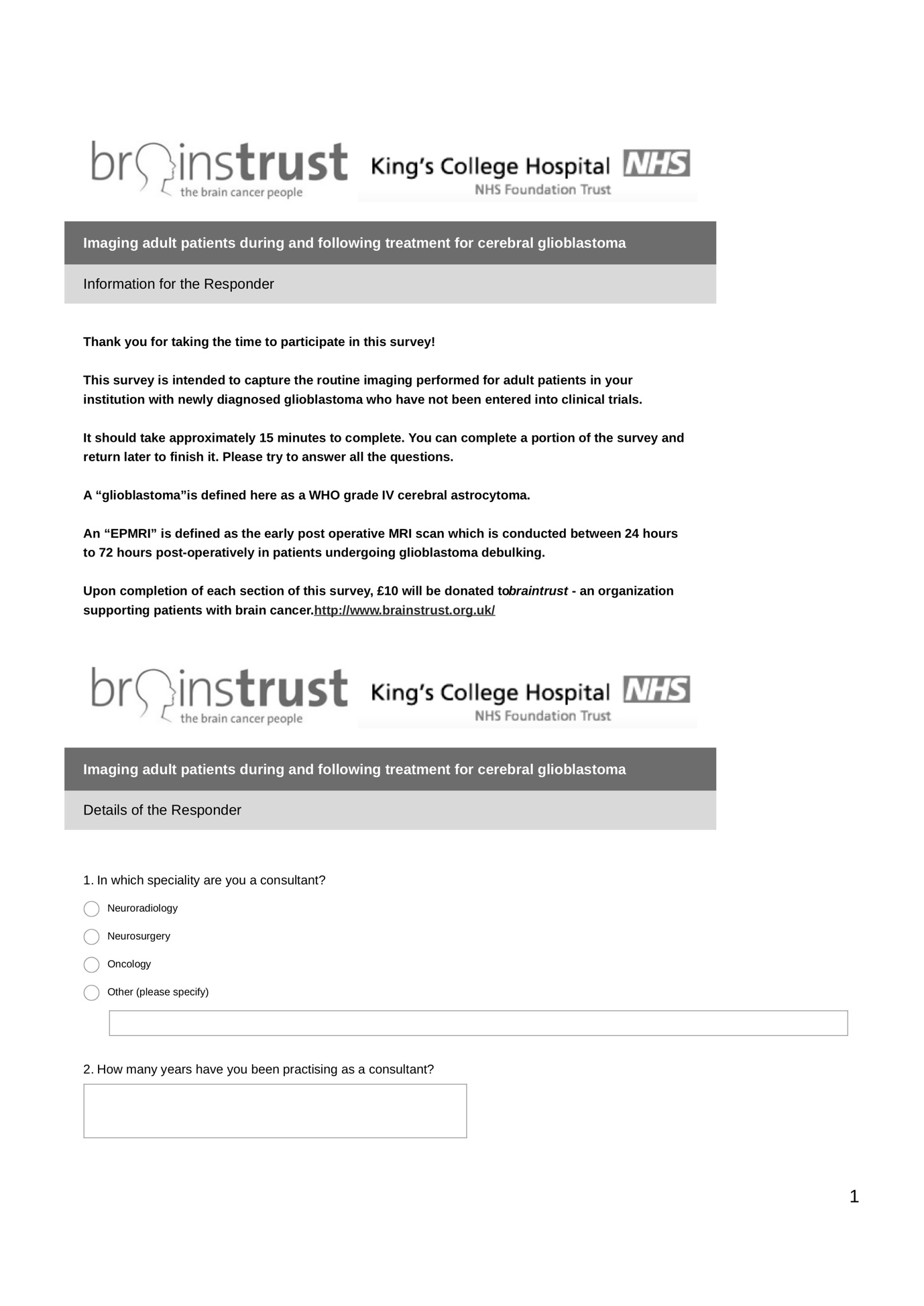
*

REDACTED FOR PEER REVIEW REVIEW

REDACTED FOR PEER REVIEW REVIEW

*
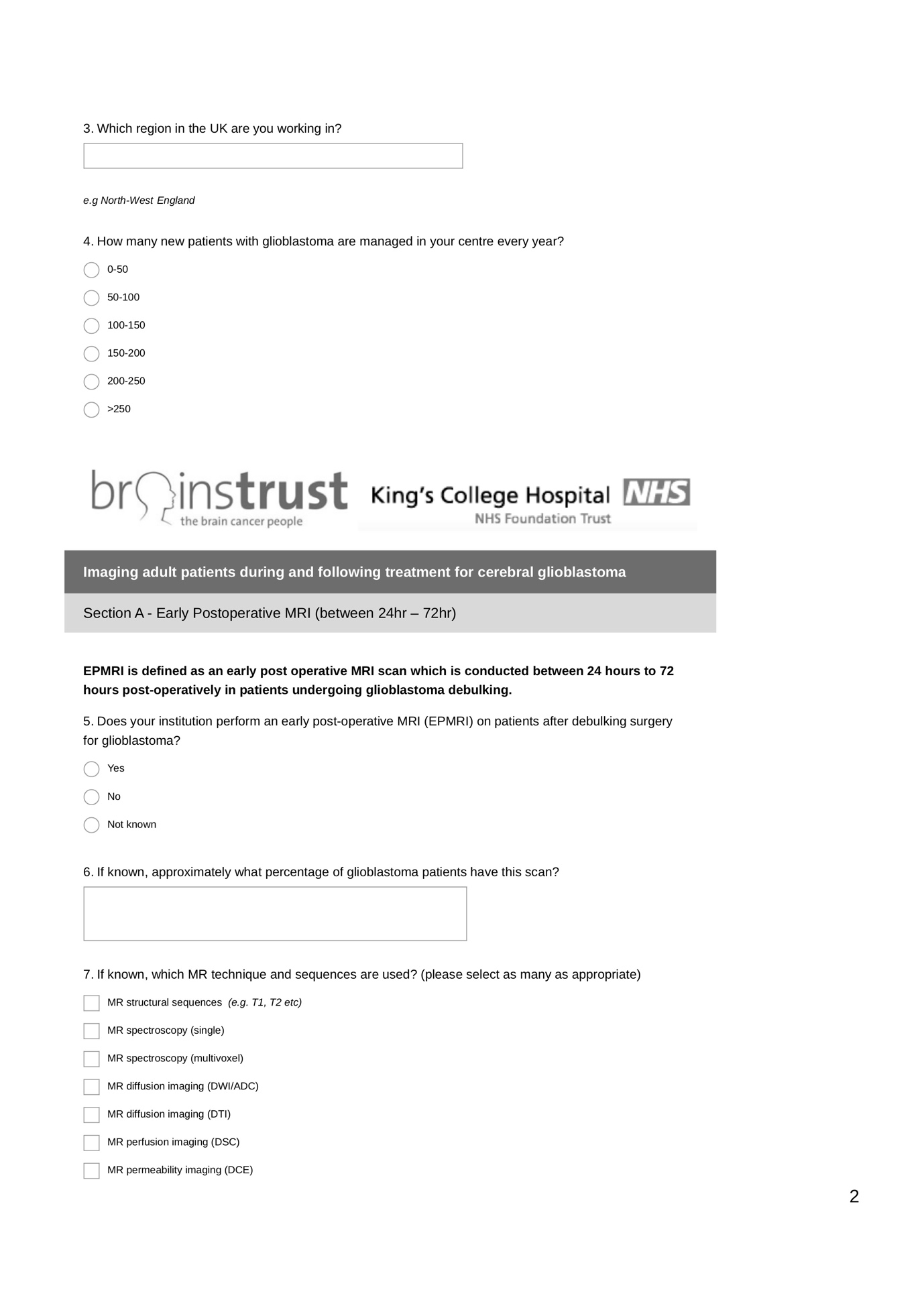
*

REDACTED FOR PEER REVIEW REVIEW

*
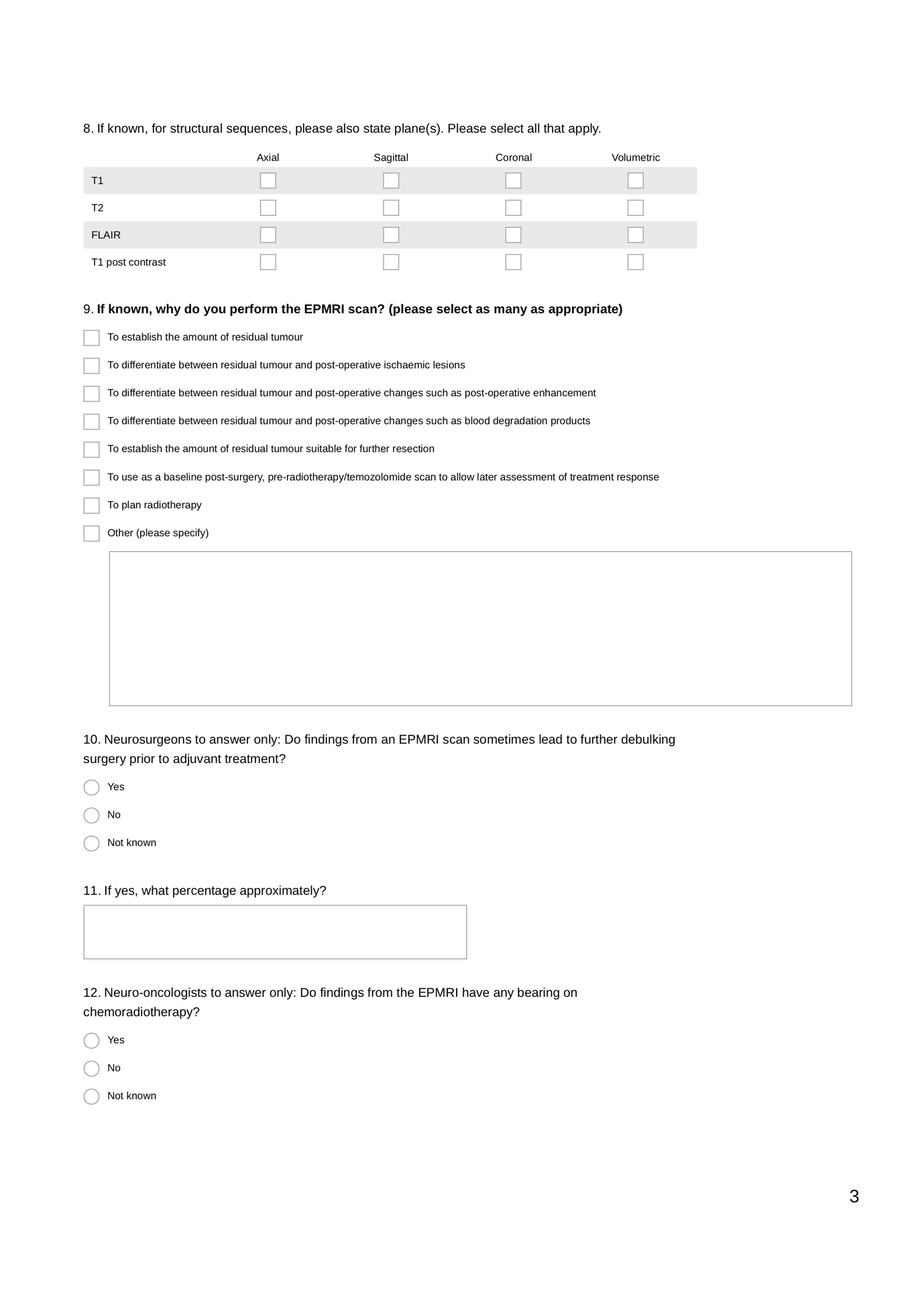
*

*
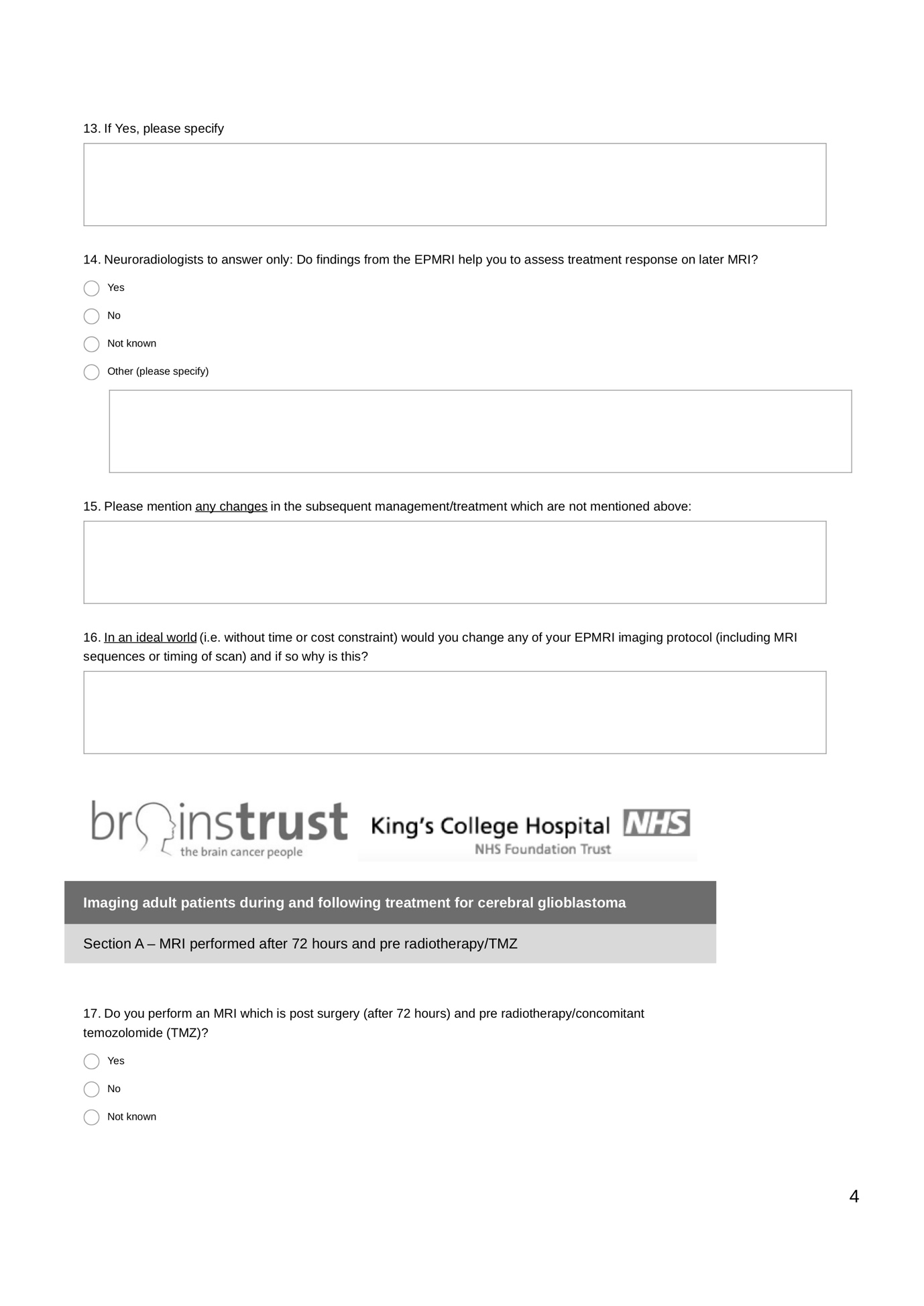
*

REDACTED FOR PEER REVIEW REVIEW

*
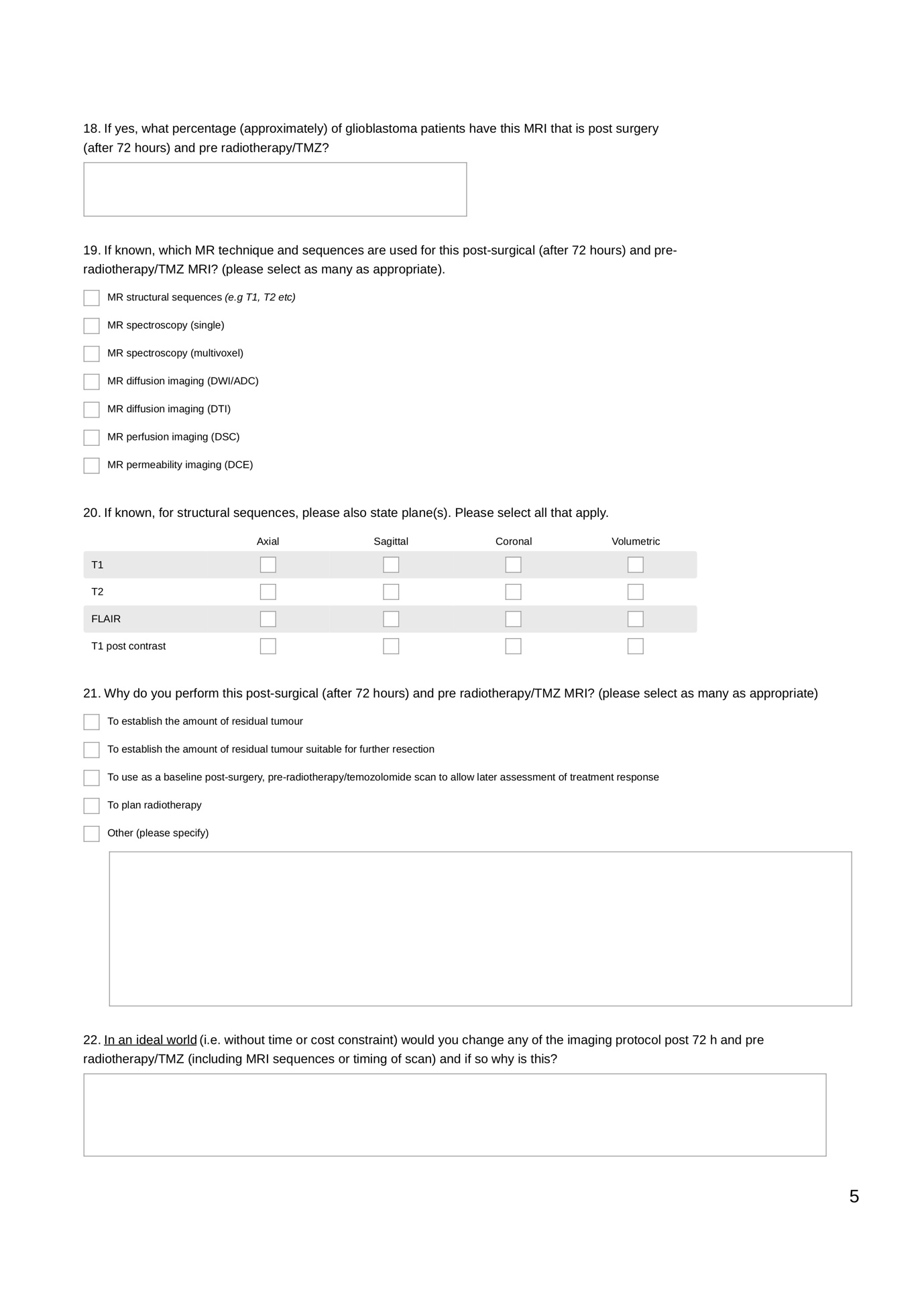
*

*
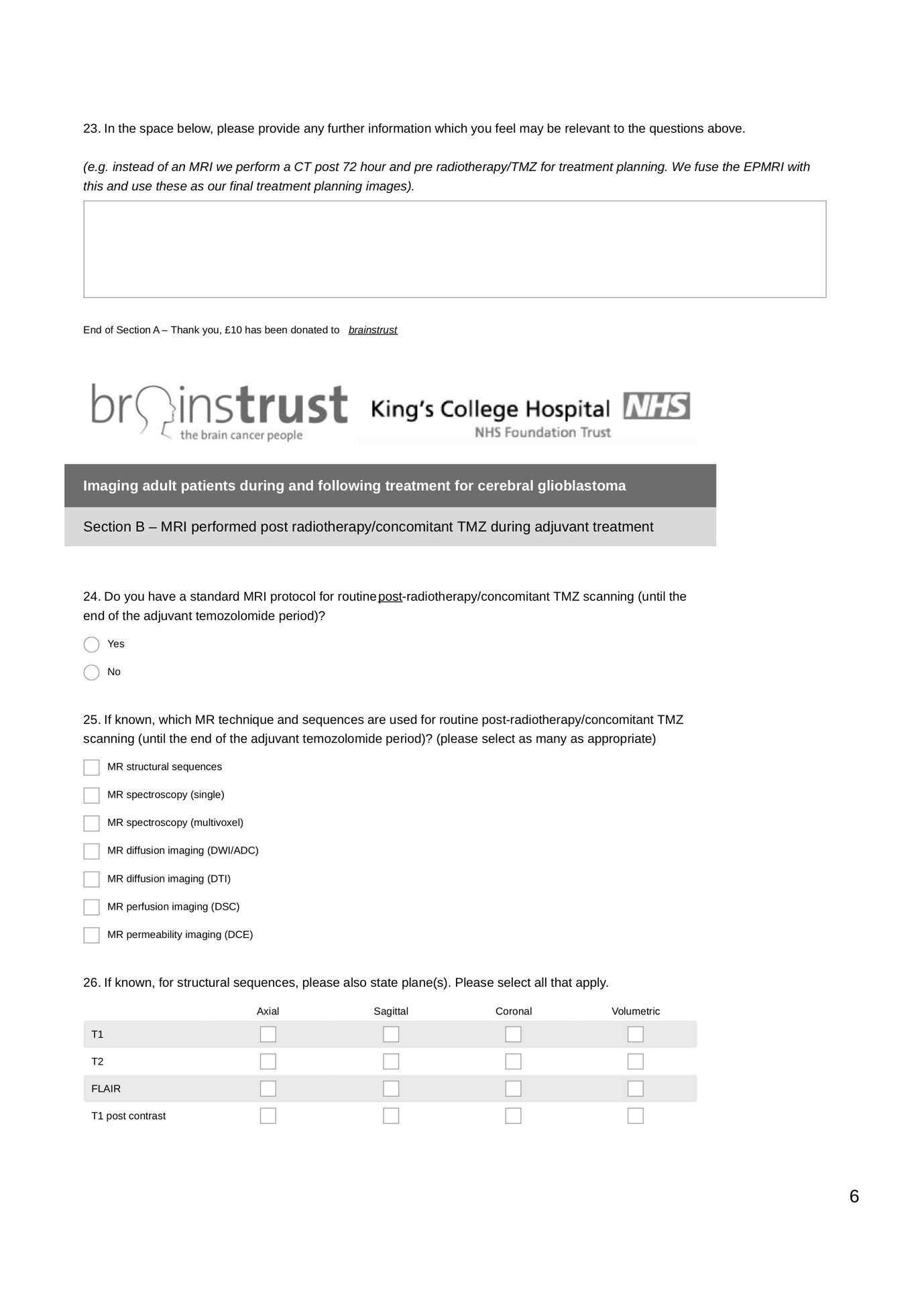
*

REDACTED FOR PEER REVIEW REVIEW

*
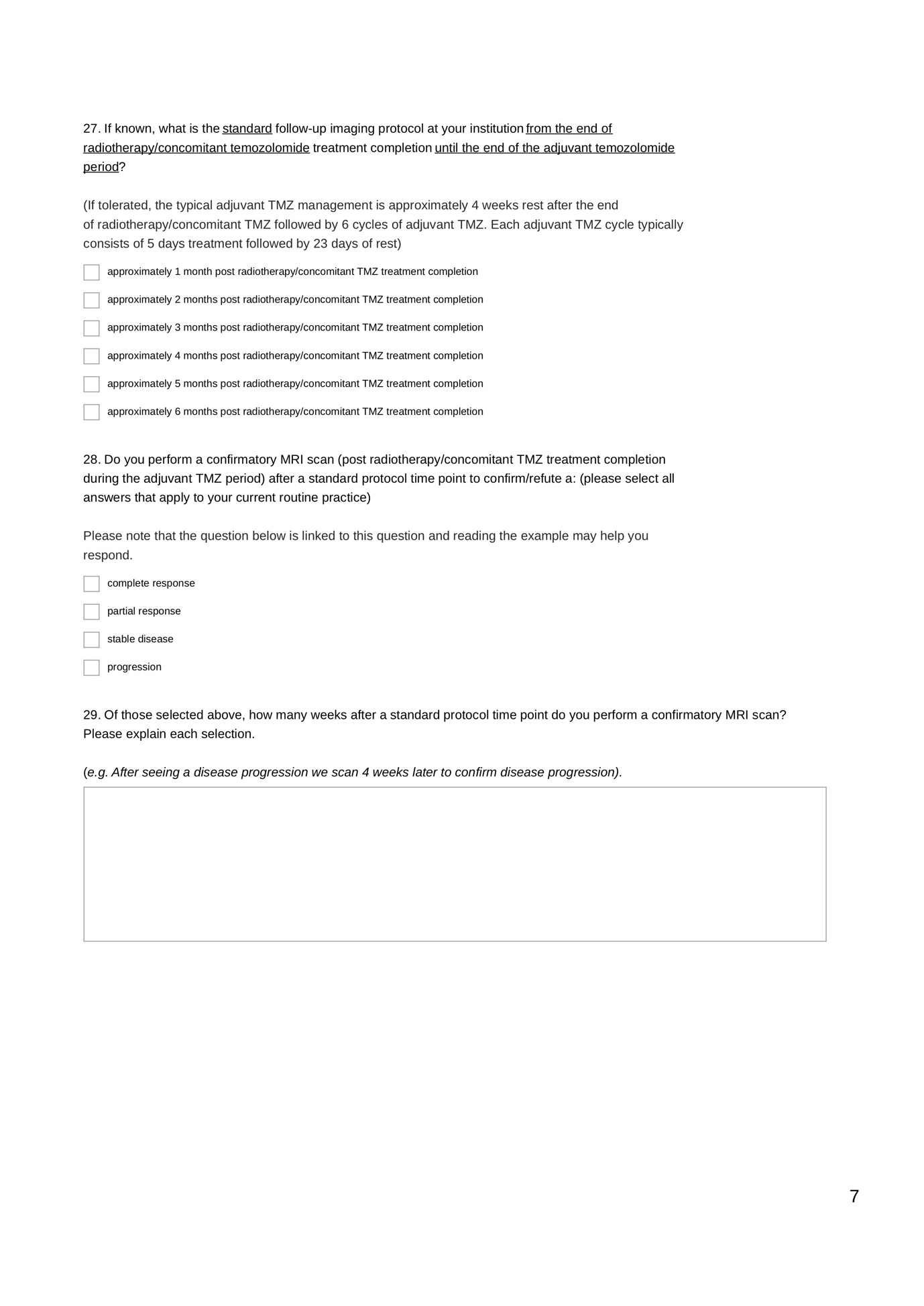
*

*
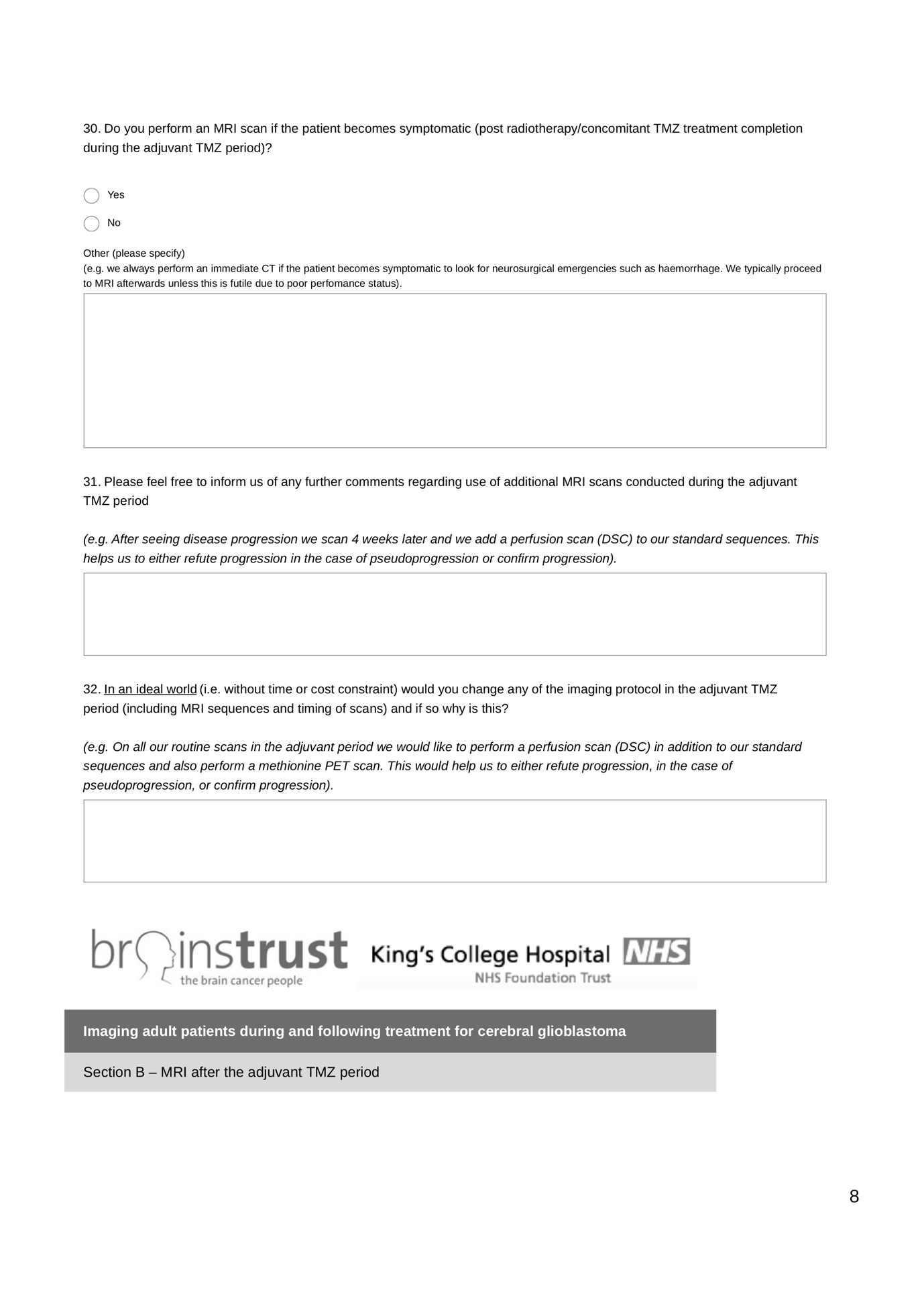
*

REDACTED FOR PEER REVIEW REVIEW

*
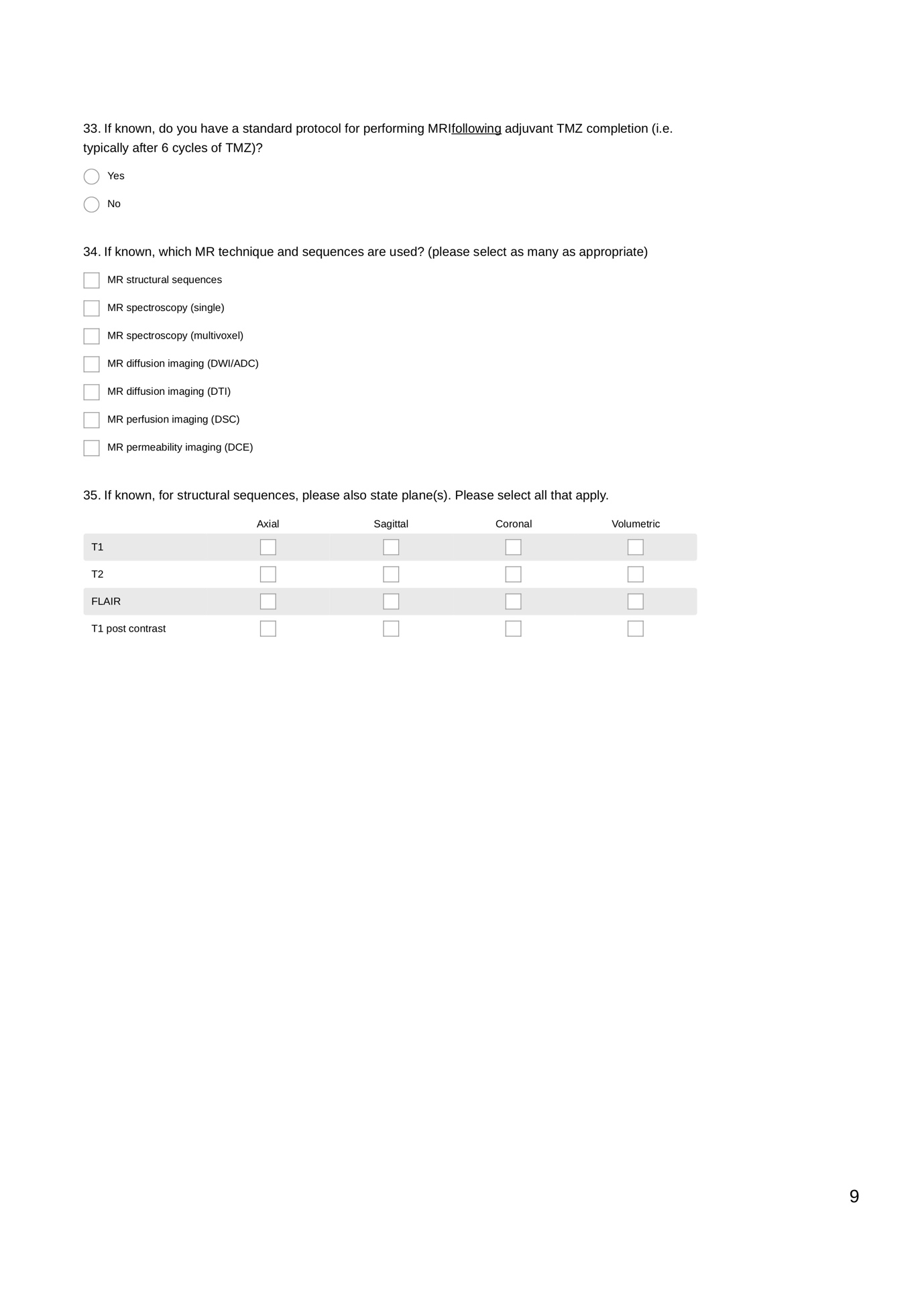
*

*
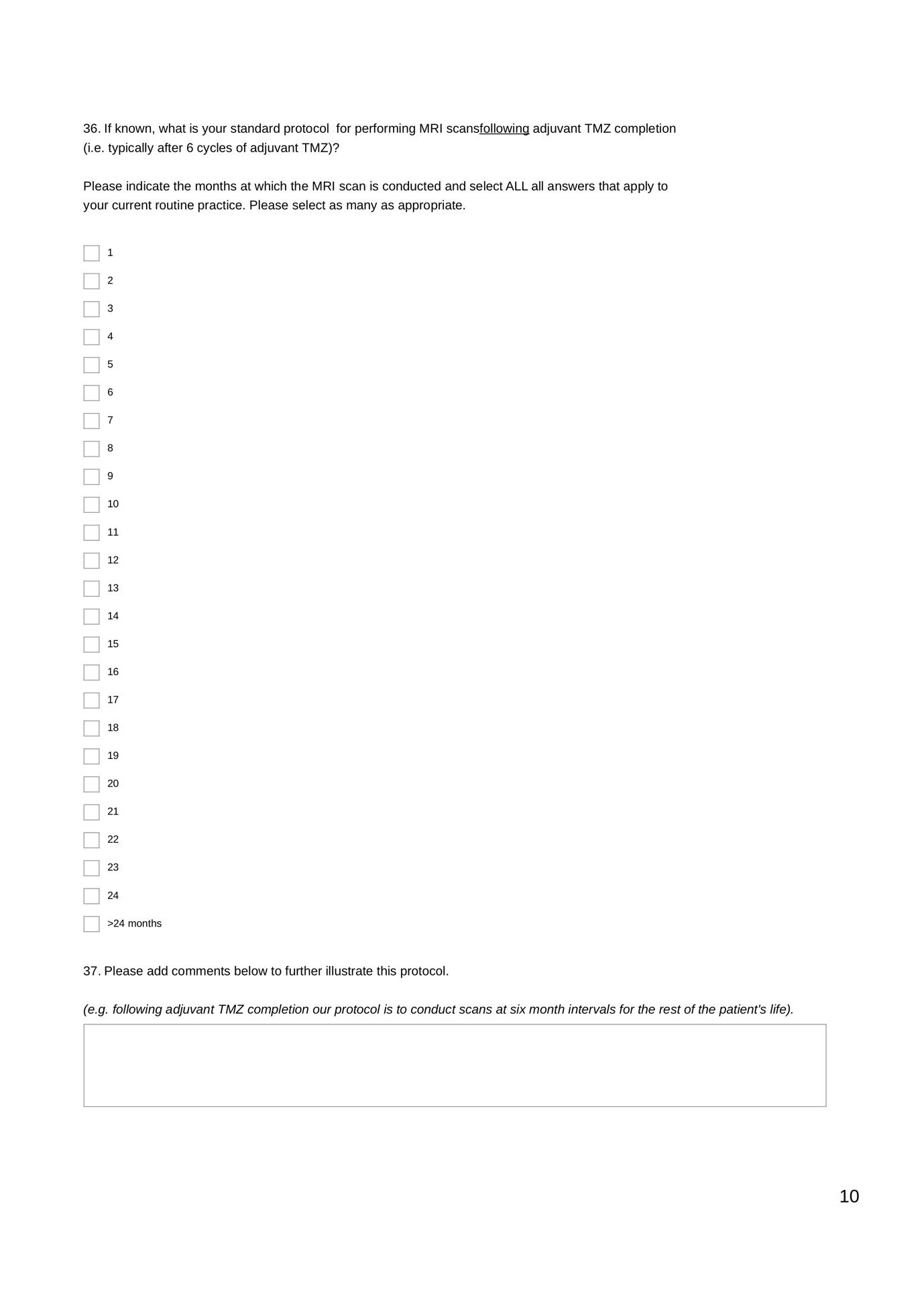
*

*
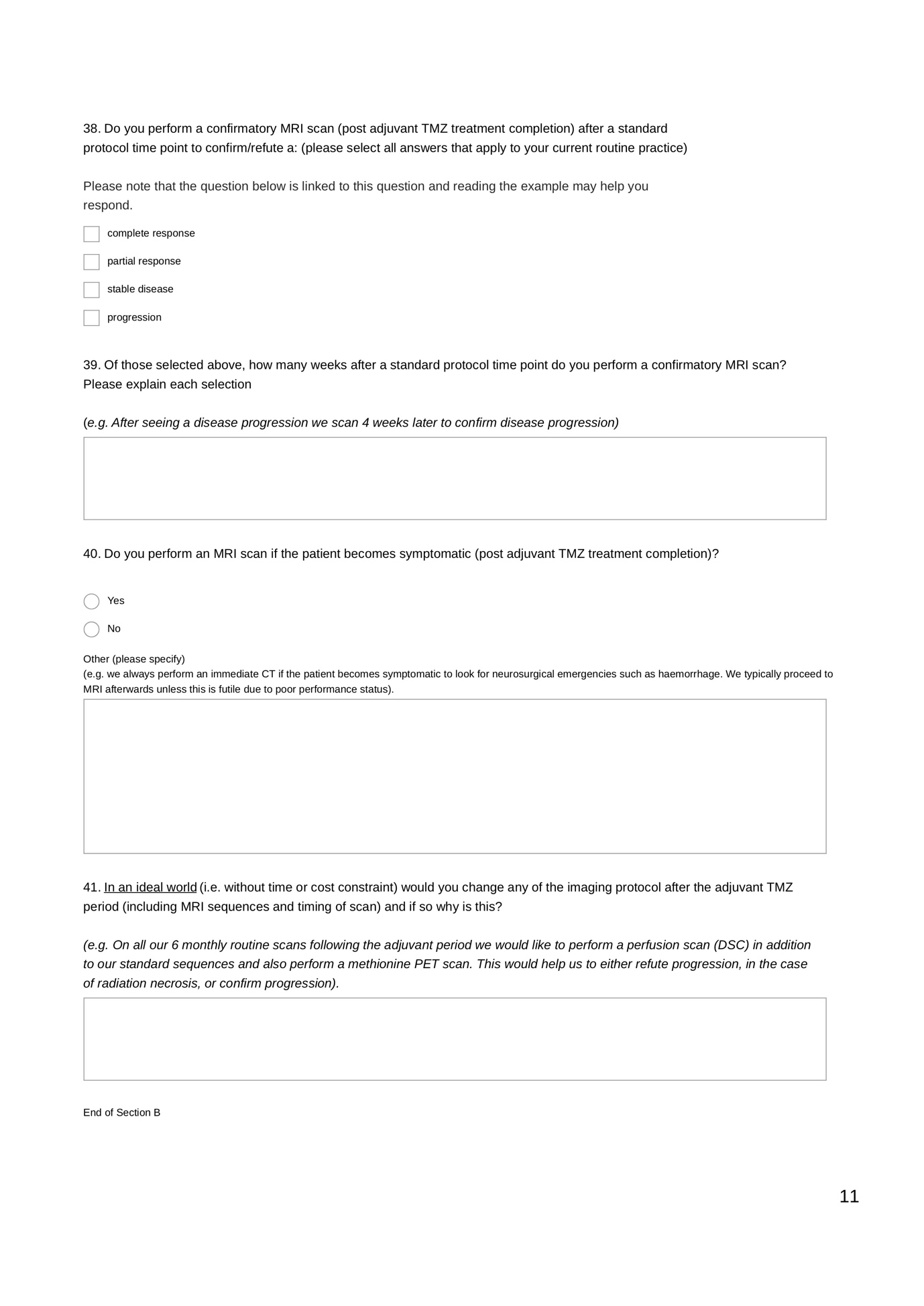
*

*
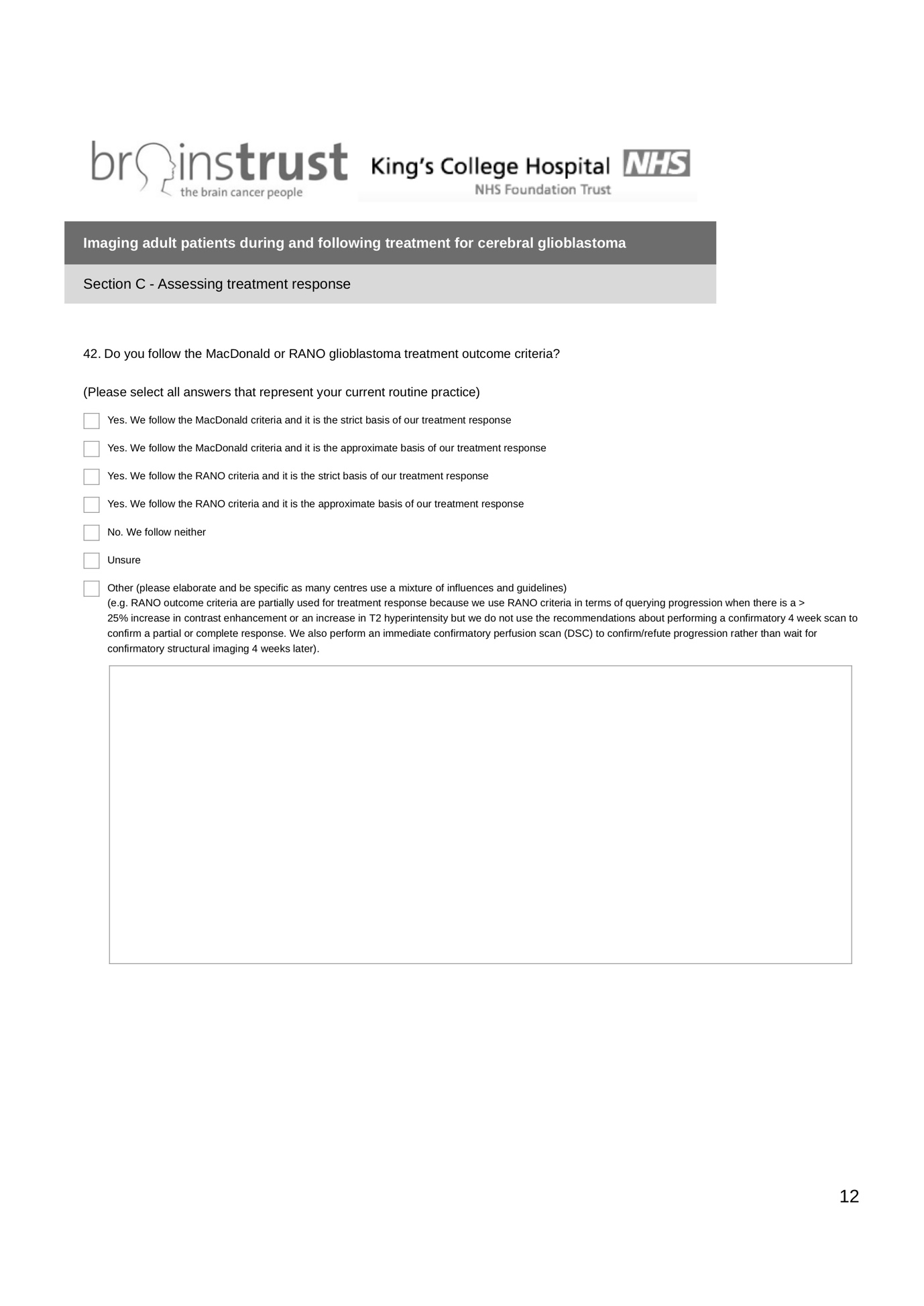
*

REDACTED FOR PEER REVIEW REVIEW

*
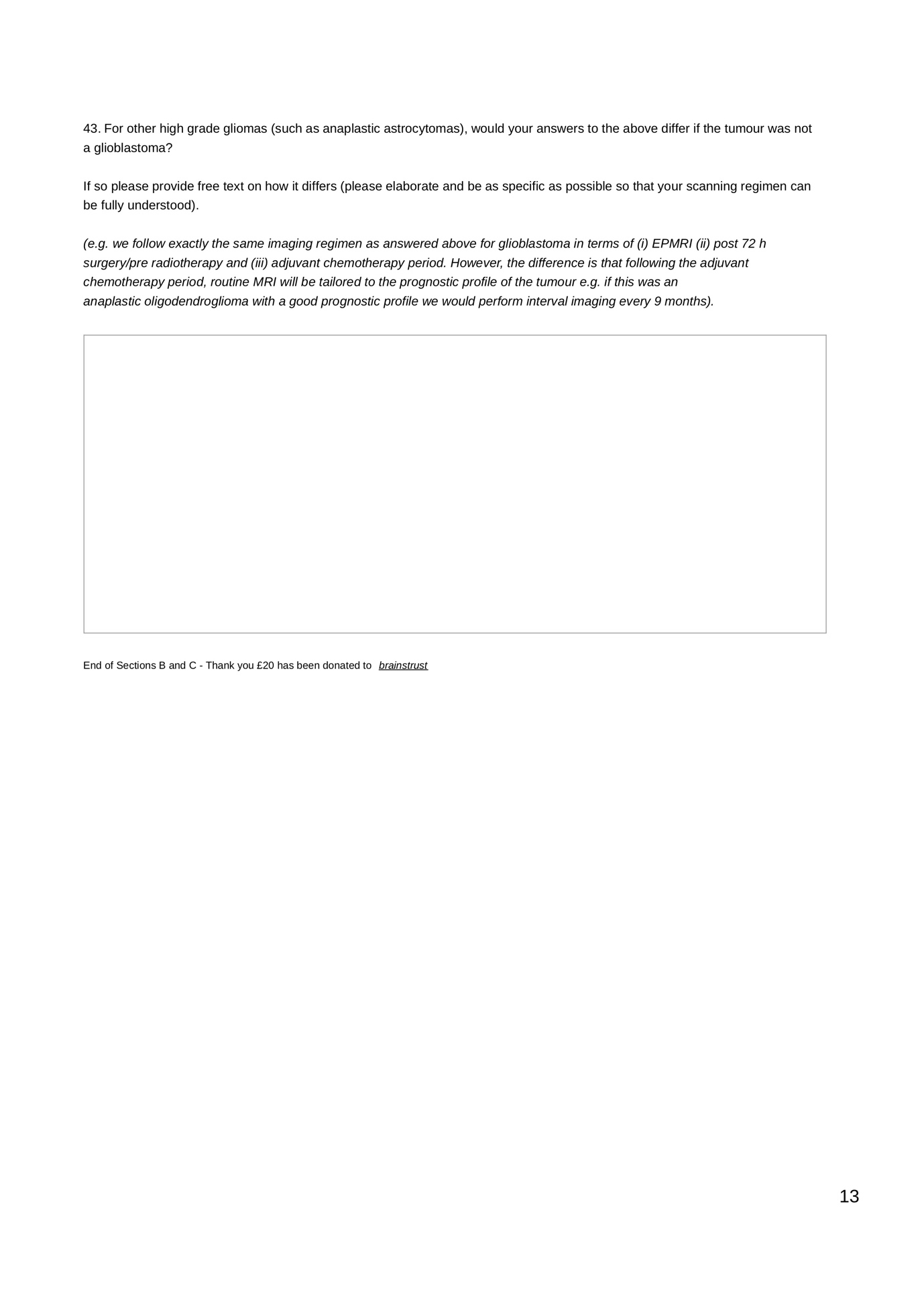
*

**Appendix 2**

**Health Economic Resource Use of Neuro-Oncology Centres**

A health economic resource use analysis is beyond the remit of the study. Nonetheless, local resource use analysis might influence the clinical decision-making mechanisms at each centre.

Public National Health Service academic centres are homogenous in terms of:

1. Overarching health policy: The UK neuro-oncology centres aim to follow best evidence according to the National Institute for Health and Care Excellence (NICE) (1). There is no definitive evidence for MRI post-operative imaging in neuro-oncology (2).
2. Overarching health assets: The UK only has 6.1 MRI systems per million population, the second lowest European country as reported by the OECD - in contrast Germany has 30.5 MRI systems per million population (3,4). MRI activity is also low at only 56.3 scans per 1,000 head of population, but just slightly under the OECD average, clearly demonstrating a very high level of utilisation. A centre’s ability to increase use alone is unlikely to bridge the capacity gap (5).

On a background of homogenous overarching health policy and assets, the local centre utilisation rates of MRI can be measured both by hours per week of operation and by patient throughput per day which are known to be variable (5). Whilst capacity can be increased by increasing equipment use, other factors will influence capacity including radiographic and other clinical staffing recruitment and retention. It may also be affected by an individual institution’s approach to the adoption of skills mix, teamworking, complexity of the examination and patient condition, together with patient referral patterns.

1. National Institute for Health and Care Excellence. https://www.nice.org.uk/about/what-we-do/our-programmes/nice-guidance/nice-guidelines. Accessed Mar 2020
2. National Institute for Health and Clinical Excellence (2018) *NICE guideline [NG99]: Brain tumours (primary) and brain metastases in adults*. National Institute for Health and Clinical Excellence, UK. Available from: https://www.nice.org.uk/guidance/ng99. Accessed Jan 2019.
3. Organisation for Economic Co-operation and Development (OECD). Magnetic resonance imaging (MRI) units 2013. <https://data.oecd.org/healtheqt/magnetic-resonance-imaging-mri-units.htm> . Accessed Mar 2020
4. Office for National Statistics (ONS). https://www.ons.gov.uk/peoplepopulationandcommunity/populationandmigration/populationestimates/bulletins/annualmidyearpopulationestimates/mid2015. Accessed Mar 2020
5. The Royal College of Radiologists. Magnetic resonance imaging (MRI) equipment, operations and planning in the NHS. London: The Royal College of Radiologists, 2017. https://www.rcr.ac.uk/sites/default/files/cib_mri_equipment_report.pdf Accessed Mar 2020

**Appendix 3**

**Imaging WHO Grade III gliomas**

Twenty centres (64%, 20/31) reported that the imaging protocol for all grade III gliomas [1] was the same as for glioblastoma. Ten centres (32%, 10/31) reported that the initial imaging protocol was the same, however, the imaging intervals were sometimes increased in comparison to the intervals used in glioblastoma. This might occur following completion of adjuvant TMZ if the prognostic profile of the tumour was favourable (e.g. 1p/19q co-deletion). The interval might also be influenced by the treatment given, in particular if it was radiotherapy alone or adjuvant chemotherapy. There was no response given for the remaining centre.

1. Louis, D.N., et al., *The 2016 World Health Organization Classification of Tumors of the Central Nervous System: a summary.* Acta Neuropatho

l, 2016. **131**(6): p. 803-20.
